# Poly[[μ_2_-1,2-bis­(diphenyl­phosphan­yl)-1,2-diethylhydrazine]-μ_4_-nitrato-μ_2_-nitrato-silver(I)]

**DOI:** 10.1107/S1600536810019094

**Published:** 2010-05-26

**Authors:** Frederik H. Kriel, Manuel A. Fernandes, Judy Coates

**Affiliations:** a Project AuTEK, Mintek, Private Bag X3015, Randburg 2125, South Africa; b Molecular Science Institute, School of Chemistry, University of the Witwatersrand, PO Wits, 2050 Johannesburg, South Africa

## Abstract

The title compound, [Ag_2_(NO_3_)_2_(C_28_H_30_N_2_P_2_)]_*n*_, crystallizes in polymeric α-helices. Three O atoms from three different nitrate ions in equatorial positions and two Ag atoms at axial positions set up a trigonal bipyramid. These units are linked by the phosphine ligands into endless helical chains that run along the *c* axis. The crystal used for the data collection was a racemic twin.

## Related literature

For related structures, see: Reddy *et al.* (1994 ▶, 1995 ▶); Hu (2000 ▶). 
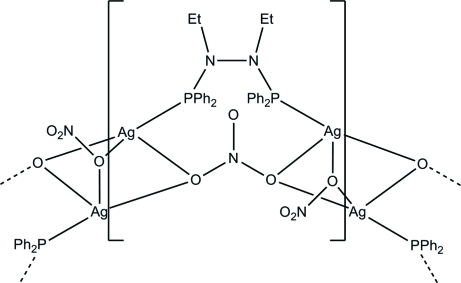


## Experimental

### 

#### Crystal data

[Ag_2_(NO_3_)_2_(C_28_H_30_N_2_P_2_)]
*M* *_r_* = 796.24
Orthorhombic, 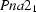

*a* = 16.332 (1) Å
*b* = 20.6486 (13) Å
*c* = 9.0164 (5) Å
*V* = 3040.6 (3) Å^3^
*Z* = 4
Mo *K*α radiation
μ = 1.44 mm^−1^
*T* = 173 K
0.22 × 0.08 × 0.07 mm

#### Data collection

Bruker SMART CCD area-detector diffractometer
Absorption correction: analytical (*SADABS*; Bruker, 1999 ▶) *T*_min_ = 0.788, *T*_max_ = 0.907
13103 measured reflections
8801 independent reflections
6217 reflections with *I* > 2σ(*I*)
*R*_int_ = 0.051

#### Refinement

*R*[*F* ^2^ > 2σ(*F* ^2^)] = 0.046
*wR*(*F* ^2^) = 0.161
*S* = 1.01
8801 reflections
380 parameters
1 restraint
H-atom parameters constrained
Δρ_max_ = 0.58 e Å^−3^
Δρ_min_ = −0.73 e Å^−3^
Absolute structure: Flack (1983 ▶), 3898 Friedel pairs
Flack parameter: 0.53 (4)

### 

Data collection: *SMART-NT* (Bruker, 1998 ▶); cell refinement: *SAINT-Plus* (Bruker, 1999 ▶); data reduction: *SAINT-Plus*; program(s) used to solve structure: *SHELXS97* (Sheldrick, 2008 ▶); program(s) used to refine structure: *SHELXL97* (Sheldrick, 2008 ▶); molecular graphics: *ORTEP-3* (Farrugia, 1997 ▶) and *Mercury* (Macrae *et al.*, 2008 ▶); software used to prepare material for publication: *WinGX* (Farrugia, 1999 ▶).

## Supplementary Material

Crystal structure: contains datablocks I, global. DOI: 10.1107/S1600536810019094/fk2018sup1.cif

Structure factors: contains datablocks I. DOI: 10.1107/S1600536810019094/fk2018Isup2.hkl

Additional supplementary materials:  crystallographic information; 3D view; checkCIF report

## Figures and Tables

**Fig. 1.**
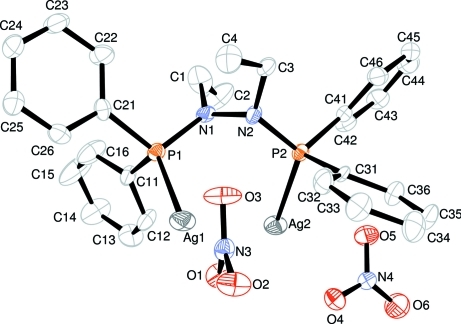
: Molecular structure of I drawn with displacement ellipsoids at the 50 % probability level. Hydrogen atoms have been omitted for clarity.

**Fig. 2.**
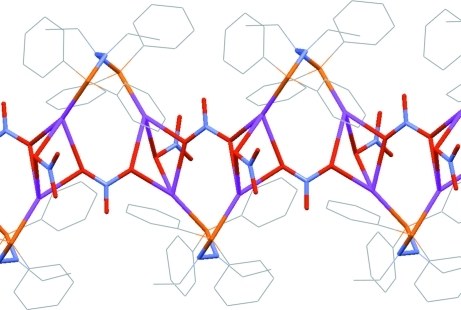
: Mercury representation of I showing the polymeric helix. Hydrogen atoms have been omitted for clarity.

## supplementary crystallographic information

### Comment

The centre of a polymeric α-helix produced by the title compound is filled by nitrate counter ions. Three oxygen atoms from three different nitrate ions occupy the equatorial positions of a trigonal bipyramid and two silver atoms are situated at the axial positions. This complex arrangement connects each silver atom of one complex to a silver atom in a neighbouring complex. The resulting α-helices (Figure 2) are packed parallel to each other and run down the c-axis of the crystal.

Polymerisation in silver nitrate complexes analogous to the title compound is commonly encountered. For example, the case of (NO_3_)Ag(dppe)Ag(NO_3_) that forms long chains with P—Ag—P units as the connecting entity (Hu, 2000). These long chains are also periodically connected by short chains of Ag—O—Ag bonds, giving rise to sheets of connected complexes. The Ag—P bond distances of 2.334 (2) Å and 2.349 (2) Å in the title compound are considerably shorter than those of (NO_3_)Ag(dppe)Ag(NO_3_) (2.41 Å and 2.42 Å). The trigonal bipyramidal structure of the title compound consists of four long Ag—O bonds in the range of 2.5 Å and two short Ag—O bonds in the range of 2.3 Å. Two longer bonds are to the same NO_3_^-^, while two sets of a short and long bond are connected to the other two nitrates, respectively. This compares to Ag—O bond lengths between 2.68 Å and 2.17 Å in (NO_3_)Ag(dppe)Ag(NO_3_). The title compound exhibits Ag—O—Ag angles in the range of 93° to 99° and O—Ag—O angles in the range of 66° to 75°.

### Experimental

Silver nitrate (100 mg, 0.59 mmol) was suspended in THF or dissolved in the minimum amount of acetonitrile. To the stirred suspension were added 0.5 equivalents of bis(diphenylphosphino)-1,2-diethylhydrazine (132 mg, 0.29 mmol) in dichloromethane (DCM) (5 ml). The suspension turned light brown. The solvent was removed *in vacuo* to afford the product as a solid (65% yield).

The title compound crystallised from a mixture of acetonitrile, ethylacetate and hexane after being left at -20 °C for two weeks.

### Refinement

The crystal studied was a racemic twin, the refined ratio of twin components being 0.53 (4) : 0.47 (4). The H atoms were positioned geometrically and allowed to ride on their respective parent atoms, with C—H = 0.93 (Ar—H) or 0.96 (CH_3_) Å, and with U_eq_ = 1.2 (Ar—H) or 1.5 (CH_3_) U_eq_(C).

### Crystal data

**Table:**

[Ag_2_(NO_3_)_2_(C_28_H_30_N_2_P_2_)]	*F*(000) = 1592
*M**_r_* = 796.24	*D*_x_ = 1.739 Mg m^−^^3^
Orthorhombic, *P**n**a*2_1_	Mo *K*α radiation, λ = 0.71073 Å
Hall symbol: P 2c -2n	Cell parameters from 3919 reflections
*a* = 16.332 (1) Å	θ = 2.3–26.5°
*b* = 20.6486 (13) Å	µ = 1.44 mm^−^^1^
*c* = 9.0164 (5) Å	*T* = 173 K
*V* = 3040.6 (3) Å^3^	Needle, colourless
*Z* = 4	0.22 × 0.08 × 0.07 mm

### Data collection

**Table:**

Bruker SMART CCD area-detector diffractometer	8801 independent reflections
Radiation source: fine-focus sealed tube	6217 reflections with *I* > 2σ(*I*)
graphite	*R*_int_ = 0.051
phi and ω scans	θ_max_ = 30.5°, θ_min_ = 1.6°
Absorption correction: analytical (*SADABS*; Bruker, 1999)	*h* = −20→23
*T*_min_ = 0.788, *T*_max_ = 0.907	*k* = −10→29
13103 measured reflections	*l* = −12→12

### Refinement

**Table:**

Refinement on *F*^2^	Secondary atom site location: difference Fourier map
Least-squares matrix: full	Hydrogen site location: inferred from neighbouring sites
*R*[*F*^2^ > 2σ(*F*^2^)] = 0.046	H-atom parameters constrained
*wR*(*F*^2^) = 0.161	*w* = 1/[σ^2^(*F*_o_^2^) + (0.0891*P*)^2^] where *P* = (*F*_o_^2^ + 2*F*_c_^2^)/3
*S* = 1.01	(Δ/σ)_max_ = 0.006
8801 reflections	Δρ_max_ = 0.58 e Å^−^^3^
380 parameters	Δρ_min_ = −0.73 e Å^−^^3^
1 restraint	Absolute structure: Flack (1983), 3898 Friedel pairs
Primary atom site location: structure-invariant direct methods	Flack parameter: 0.53 (4)

### Special details

**Table:**

Experimental. Intensity data were collected on a Bruker SMART1K CCD area detector diffractometer with graphite monochromated Mo Kα radiation (40 kV, 40 mA). The collection method involved ω-scans of width 0.3°. Data reduction was carried out using the program SAINT+ (Bruker, 1999) and face indexed absorption corrections were made using the program SAINT+ SADABS.^1^H NMR: (*d*-DMSO, 300 MHz) δ_H_7.79 (bs, Arom), 7.64 (bs, Arom), 7.54 (bs, Arom), 3.19 (m, CH_2_CH_3_), 0.57 (t, ^3^*J*(^1^H-^1^H) *= *6.6 Hz, CH_2_CH_3_).^13^C NMR: (CDCl_3_, 100.6 MHz) δ_C_ 135.8 (s,Arom), 134.0 (Arom), 131.2 (m, Arom), 128.2 (s, Arom), *ethyl signals could not be observed*. ^31^P NMR: (*d*-DMSO, 121 MHz) δ_P_ 77.14 (d, ^1^*J *(^107/109^Ag-^31^P)*= *782.9 Hz). MS: 733 (5 %, M - NO_3_), 563 (7 %, Ligand + Ag). EA: Calc: (Ag_2_P_2_N_4_O_6_C_28_H_30_) C 42.24% H 3.80% N 7.04%. Found: C 41.48% H 3.95% N 6.77%. MP: 182 – 183 °C.
Geometry. All esds (except the esd in the dihedral angle between two l.s. planes) are estimated using the full covariance matrix. The cell esds are taken into account individually in the estimation of esds in distances, angles and torsion angles; correlations between esds in cell parameters are only used when they are defined by crystal symmetry. An approximate (isotropic) treatment of cell esds is used for estimating esds involving l.s. planes.
Refinement. Refinement of F^2^ against ALL reflections. The weighted R-factor wR and goodness of fit S are based on F^2^, conventional R-factors R are based on F, with F set to zero for negative F^2^. The threshold expression of F^2^ > 2sigma(F^2^) is used only for calculating R-factors(gt) etc. and is not relevant to the choice of reflections for refinement. R-factors based on F^2^ are statistically about twice as large as those based on F, and R- factors based on ALL data will be even larger.

### Fractional atomic coordinates and isotropic or equivalent isotropic displacement parameters (Å^2^)

**Table:**

	*x*	*y*	*z*	*U*_iso_*/*U*_eq_	
C1	0.1939 (6)	0.2253 (5)	0.8345 (9)	0.044 (2)	
H1A	0.1595	0.2635	0.8325	0.053*	
H1B	0.2486	0.2394	0.8613	0.053*	
C2	0.1976 (7)	0.1994 (4)	0.6833 (12)	0.057 (3)	
H2A	0.2186	0.2320	0.6175	0.086*	
H2B	0.1437	0.1870	0.6520	0.086*	
H2C	0.2330	0.1623	0.6813	0.086*	
C3	0.2810 (5)	0.1667 (3)	1.1170 (9)	0.0296 (16)	
H3A	0.3098	0.2001	1.0621	0.036*	
H3B	0.3203	0.1334	1.1430	0.036*	
C4	0.2457 (6)	0.1971 (4)	1.2633 (9)	0.042 (2)	
H4A	0.2897	0.2147	1.3213	0.063*	
H4B	0.2180	0.1642	1.3195	0.063*	
H4C	0.2078	0.2309	1.2387	0.063*	
C11	0.0019 (4)	0.1970 (3)	0.8645 (8)	0.0258 (14)	
C12	−0.0291 (4)	0.1410 (3)	0.7955 (10)	0.0324 (16)	
H12	−0.0198	0.1007	0.8387	0.039*	
C13	−0.0742 (5)	0.1446 (4)	0.6624 (11)	0.043 (2)	
H13	−0.0899	0.1069	0.6140	0.051*	
C14	−0.0947 (7)	0.2036 (5)	0.6040 (12)	0.055 (3)	
H14	−0.1265	0.2066	0.5187	0.066*	
C15	−0.0663 (8)	0.2597 (4)	0.6771 (14)	0.073 (4)	
H15	−0.0794	0.3003	0.6394	0.087*	
C16	−0.0200 (6)	0.2550 (4)	0.8018 (11)	0.050 (2)	
H16	−0.0022	0.2930	0.8469	0.061*	
C21	0.0544 (4)	0.2647 (3)	1.1204 (7)	0.0232 (14)	
C22	0.1097 (5)	0.3142 (4)	1.1123 (9)	0.0343 (17)	
H22	0.1579	0.3089	1.0586	0.041*	
C23	0.0939 (5)	0.3735 (4)	1.1854 (11)	0.0387 (18)	
H23	0.1300	0.4080	1.1749	0.046*	
C24	0.0265 (5)	0.3800 (4)	1.2701 (9)	0.0364 (18)	
H24	0.0169	0.4190	1.3188	0.044*	
C25	−0.0285 (5)	0.3300 (4)	1.2860 (10)	0.0372 (17)	
H25	−0.0741	0.3345	1.3469	0.045*	
C26	−0.0146 (4)	0.2725 (4)	1.2090 (9)	0.0339 (18)	
H26	−0.0521	0.2387	1.2170	0.041*	
C31	0.2553 (4)	0.0102 (3)	1.0819 (8)	0.0197 (13)	
C32	0.2246 (5)	0.0190 (4)	1.2244 (9)	0.0330 (17)	
H32	0.1968	0.0569	1.2486	0.040*	
C33	0.2358 (5)	−0.0291 (4)	1.3306 (8)	0.0369 (18)	
H33	0.2176	−0.0223	1.4271	0.044*	
C34	0.2745 (5)	−0.0883 (4)	1.2934 (11)	0.0424 (19)	
H34	0.2799	−0.1212	1.3632	0.051*	
C35	0.3038 (5)	−0.0959 (4)	1.1521 (11)	0.040 (2)	
H35	0.3317	−0.1337	1.1282	0.048*	
C36	0.2933 (5)	−0.0499 (4)	1.0457 (9)	0.0317 (16)	
H36	0.3109	−0.0577	0.9493	0.038*	
C41	0.3281 (4)	0.0657 (3)	0.8277 (7)	0.0225 (14)	
C42	0.3247 (4)	0.0570 (3)	0.6732 (9)	0.0300 (14)	
H42	0.2741	0.0544	0.6261	0.036*	
C43	0.3958 (5)	0.0522 (4)	0.5907 (9)	0.0341 (17)	
H43	0.3932	0.0445	0.4891	0.041*	
C44	0.4710 (5)	0.0589 (4)	0.6604 (12)	0.0411 (19)	
H44	0.5186	0.0585	0.6037	0.049*	
C45	0.4768 (4)	0.0661 (3)	0.8130 (10)	0.0331 (16)	
H45	0.5276	0.0679	0.8595	0.040*	
C46	0.4062 (5)	0.0705 (3)	0.8933 (9)	0.0301 (15)	
H46	0.4098	0.0770	0.9951	0.036*	
N1	0.1635 (4)	0.1824 (3)	0.9553 (6)	0.0232 (12)	
N2	0.2181 (3)	0.1384 (3)	1.0214 (6)	0.0222 (11)	
N3	0.0603 (4)	0.0379 (3)	1.4801 (6)	0.0229 (12)	
N4	0.1688 (4)	−0.1152 (3)	0.6966 (9)	0.0337 (15)	
O1	0.0241 (3)	0.0220 (2)	1.3584 (5)	0.0275 (11)	
O2	0.0429 (3)	0.0039 (3)	1.5915 (5)	0.0341 (12)	
O3	0.1085 (4)	0.0816 (3)	1.4847 (6)	0.0413 (15)	
O4	0.1100 (3)	−0.1018 (3)	0.7843 (6)	0.0352 (12)	
O5	0.2198 (3)	−0.0715 (3)	0.6727 (8)	0.0440 (13)	
O6	0.1766 (4)	−0.1688 (3)	0.6396 (12)	0.077 (3)	
P1	0.06706 (11)	0.18681 (8)	1.02502 (19)	0.0209 (3)	
P2	0.23174 (10)	0.06614 (8)	0.93328 (19)	0.0200 (3)	
Ag1	0.03083 (3)	0.09899 (3)	1.17410 (7)	0.03480 (15)	
Ag2	0.12155 (3)	0.02110 (3)	0.80136 (7)	0.03238 (14)	

### Atomic displacement parameters (Å^2^)

**Table:**

	*U*^11^	*U*^22^	*U*^33^	*U*^12^	*U*^13^	*U*^23^
C1	0.044 (5)	0.057 (5)	0.032 (5)	0.005 (4)	0.013 (4)	0.017 (4)
C2	0.101 (8)	0.033 (4)	0.039 (5)	−0.004 (5)	0.012 (6)	−0.003 (4)
C3	0.030 (4)	0.019 (3)	0.040 (4)	0.002 (3)	−0.007 (3)	−0.009 (3)
C4	0.048 (5)	0.047 (5)	0.031 (4)	0.004 (4)	−0.006 (3)	−0.009 (4)
C11	0.029 (3)	0.019 (3)	0.029 (3)	0.006 (3)	−0.001 (3)	−0.001 (3)
C12	0.044 (4)	0.018 (3)	0.035 (4)	0.005 (3)	−0.013 (4)	0.000 (3)
C13	0.040 (4)	0.052 (5)	0.037 (4)	−0.004 (4)	−0.015 (4)	−0.014 (4)
C14	0.078 (7)	0.044 (5)	0.042 (5)	−0.004 (5)	−0.032 (5)	0.007 (4)
C15	0.127 (10)	0.026 (4)	0.065 (6)	0.006 (5)	−0.053 (8)	0.016 (5)
C16	0.078 (6)	0.027 (4)	0.047 (5)	−0.002 (4)	−0.024 (6)	0.007 (4)
C21	0.023 (3)	0.028 (3)	0.019 (3)	0.007 (3)	0.001 (2)	0.003 (3)
C22	0.038 (4)	0.032 (4)	0.033 (4)	−0.009 (3)	0.012 (3)	−0.001 (3)
C23	0.045 (4)	0.026 (4)	0.045 (5)	−0.010 (3)	0.005 (4)	−0.008 (4)
C24	0.037 (4)	0.031 (4)	0.041 (5)	0.003 (3)	0.000 (3)	−0.012 (3)
C25	0.037 (4)	0.032 (4)	0.042 (5)	0.004 (3)	0.007 (4)	−0.005 (4)
C26	0.027 (4)	0.034 (4)	0.041 (5)	−0.006 (3)	0.009 (3)	−0.002 (3)
C31	0.021 (3)	0.009 (3)	0.030 (3)	0.002 (2)	−0.003 (3)	−0.002 (2)
C32	0.035 (4)	0.031 (4)	0.032 (4)	0.007 (3)	0.009 (3)	−0.004 (3)
C33	0.049 (5)	0.038 (4)	0.024 (4)	0.003 (3)	−0.004 (3)	0.009 (3)
C34	0.047 (5)	0.042 (4)	0.038 (4)	0.012 (4)	−0.014 (4)	0.004 (4)
C35	0.046 (4)	0.020 (3)	0.054 (6)	0.009 (3)	−0.005 (4)	0.008 (3)
C36	0.040 (4)	0.024 (3)	0.031 (4)	0.007 (3)	0.002 (3)	0.003 (3)
C41	0.023 (3)	0.016 (3)	0.029 (4)	0.001 (2)	0.000 (3)	0.001 (2)
C42	0.037 (4)	0.022 (3)	0.031 (3)	−0.005 (3)	0.004 (4)	−0.005 (3)
C43	0.041 (4)	0.034 (4)	0.027 (4)	−0.005 (3)	0.010 (3)	0.004 (3)
C44	0.038 (4)	0.033 (4)	0.052 (5)	−0.002 (3)	0.019 (4)	−0.005 (4)
C45	0.025 (3)	0.025 (3)	0.049 (5)	−0.001 (3)	0.000 (4)	0.000 (4)
C46	0.030 (4)	0.027 (3)	0.034 (4)	−0.003 (3)	0.001 (3)	−0.002 (3)
N1	0.027 (3)	0.016 (3)	0.026 (3)	−0.004 (2)	0.007 (2)	0.007 (2)
N2	0.025 (3)	0.017 (3)	0.025 (3)	0.004 (2)	−0.004 (2)	−0.002 (2)
N3	0.026 (3)	0.022 (3)	0.021 (3)	0.003 (2)	0.001 (2)	0.004 (2)
N4	0.027 (3)	0.024 (3)	0.050 (4)	−0.001 (2)	−0.001 (3)	0.002 (3)
O1	0.035 (3)	0.032 (3)	0.015 (2)	−0.002 (2)	−0.0068 (19)	−0.002 (2)
O2	0.041 (3)	0.044 (3)	0.017 (2)	−0.014 (3)	−0.005 (2)	0.011 (2)
O3	0.050 (4)	0.042 (3)	0.032 (3)	−0.030 (3)	0.002 (2)	−0.003 (2)
O4	0.028 (3)	0.040 (3)	0.038 (3)	0.000 (2)	−0.004 (2)	0.001 (3)
O5	0.042 (3)	0.037 (3)	0.053 (3)	−0.007 (2)	0.008 (3)	0.016 (3)
O6	0.052 (4)	0.039 (4)	0.140 (9)	0.000 (3)	0.005 (5)	−0.036 (5)
P1	0.0242 (8)	0.0176 (7)	0.0208 (8)	0.0003 (6)	0.0012 (7)	0.0031 (6)
P2	0.0233 (8)	0.0177 (7)	0.0189 (7)	0.0008 (6)	−0.0012 (7)	−0.0023 (6)
Ag1	0.0332 (3)	0.0326 (3)	0.0385 (3)	−0.0076 (2)	0.0000 (3)	0.0181 (3)
Ag2	0.0332 (3)	0.0337 (3)	0.0302 (3)	−0.0068 (2)	−0.0103 (3)	−0.0043 (3)

### Geometric parameters (Å, °)

**Table:**

C1—C2	1.466 (13)	C32—H32	0.9300
C1—N1	1.489 (9)	C33—C34	1.416 (11)
C1—H1A	0.9700	C33—H33	0.9300
C1—H1B	0.9700	C34—C35	1.370 (14)
C2—H2A	0.9600	C34—H34	0.9300
C2—H2B	0.9600	C35—C36	1.361 (11)
C2—H2C	0.9600	C35—H35	0.9300
C3—N2	1.464 (9)	C36—H36	0.9300
C3—C4	1.570 (11)	C41—C42	1.406 (10)
C3—H3A	0.9700	C41—C46	1.409 (10)
C3—H3B	0.9700	C41—P2	1.839 (7)
C4—H4A	0.9600	C42—C43	1.383 (10)
C4—H4B	0.9600	C42—H42	0.9300
C4—H4C	0.9600	C43—C44	1.387 (12)
C11—C16	1.373 (11)	C43—H43	0.9300
C11—C12	1.408 (10)	C44—C45	1.387 (13)
C11—P1	1.808 (8)	C44—H44	0.9300
C12—C13	1.410 (12)	C45—C46	1.364 (11)
C12—H12	0.9300	C45—H45	0.9300
C13—C14	1.367 (13)	C46—H46	0.9300
C13—H13	0.9300	N1—N2	1.406 (8)
C14—C15	1.412 (14)	N1—P1	1.698 (6)
C14—H14	0.9300	N2—P2	1.704 (6)
C15—C16	1.359 (14)	N3—O3	1.197 (7)
C15—H15	0.9300	N3—O2	1.258 (7)
C16—H16	0.9300	N3—O1	1.288 (8)
C21—C22	1.365 (10)	N4—O6	1.227 (9)
C21—C26	1.391 (10)	N4—O5	1.247 (8)
C21—P1	1.836 (7)	N4—O4	1.275 (9)
C22—C23	1.414 (11)	O1—Ag1	2.302 (5)
C22—H22	0.9300	O1—Ag2^i^	2.592 (5)
C23—C24	1.347 (12)	O2—Ag2^ii^	2.314 (5)
C23—H23	0.9300	O2—Ag1^i^	2.553 (5)
C24—C25	1.376 (11)	O4—Ag1^iii^	2.506 (5)
C24—H24	0.9300	O4—Ag2	2.549 (5)
C25—C26	1.394 (11)	P1—Ag1	2.3335 (18)
C25—H25	0.9300	P2—Ag2	2.3492 (18)
C26—H26	0.9300	Ag1—O4^i^	2.506 (5)
C31—C32	1.392 (10)	Ag1—O2^iii^	2.553 (5)
C31—C36	1.426 (9)	Ag2—O2^iv^	2.314 (5)
C31—P2	1.810 (7)	Ag2—O1^iii^	2.592 (5)
C32—C33	1.392 (10)		
			
C2—C1—N1	118.5 (8)	C33—C34—H34	120.8
C2—C1—H1A	107.7	C36—C35—C34	122.1 (8)
N1—C1—H1A	107.7	C36—C35—H35	118.9
C2—C1—H1B	107.7	C34—C35—H35	118.9
N1—C1—H1B	107.7	C35—C36—C31	120.1 (8)
H1A—C1—H1B	107.1	C35—C36—H36	119.9
C1—C2—H2A	109.5	C31—C36—H36	119.9
C1—C2—H2B	109.5	C42—C41—C46	117.4 (6)
H2A—C2—H2B	109.5	C42—C41—P2	118.6 (5)
C1—C2—H2C	109.5	C46—C41—P2	123.9 (5)
H2A—C2—H2C	109.5	C43—C42—C41	120.6 (7)
H2B—C2—H2C	109.5	C43—C42—H42	119.7
N2—C3—C4	113.4 (6)	C41—C42—H42	119.7
N2—C3—H3A	108.9	C42—C43—C44	119.5 (7)
C4—C3—H3A	108.9	C42—C43—H43	120.2
N2—C3—H3B	108.9	C44—C43—H43	120.2
C4—C3—H3B	108.9	C43—C44—C45	121.4 (7)
H3A—C3—H3B	107.7	C43—C44—H44	119.3
C3—C4—H4A	109.5	C45—C44—H44	119.3
C3—C4—H4B	109.5	C46—C45—C44	118.4 (7)
H4A—C4—H4B	109.5	C46—C45—H45	120.8
C3—C4—H4C	109.5	C44—C45—H45	120.8
H4A—C4—H4C	109.5	C45—C46—C41	122.5 (7)
H4B—C4—H4C	109.5	C45—C46—H46	118.7
C16—C11—C12	116.2 (7)	C41—C46—H46	118.7
C16—C11—P1	125.8 (6)	N2—N1—C1	118.9 (6)
C12—C11—P1	118.1 (5)	N2—N1—P1	117.7 (4)
C11—C12—C13	121.3 (7)	C1—N1—P1	123.3 (5)
C11—C12—H12	119.3	N1—N2—C3	115.9 (5)
C13—C12—H12	119.3	N1—N2—P2	116.8 (4)
C14—C13—C12	120.2 (8)	C3—N2—P2	122.2 (4)
C14—C13—H13	119.9	O3—N3—O2	122.8 (6)
C12—C13—H13	119.9	O3—N3—O1	121.5 (6)
C13—C14—C15	118.1 (8)	O2—N3—O1	115.7 (6)
C13—C14—H14	121.0	O6—N4—O5	120.8 (7)
C15—C14—H14	121.0	O6—N4—O4	122.3 (7)
C16—C15—C14	120.7 (8)	O5—N4—O4	116.9 (6)
C16—C15—H15	119.6	N3—O1—Ag1	114.6 (4)
C14—C15—H15	119.6	N3—O1—Ag2^i^	132.6 (4)
C15—C16—C11	123.2 (8)	Ag1—O1—Ag2^i^	97.89 (17)
C15—C16—H16	118.4	N3—O2—Ag2^ii^	116.2 (4)
C11—C16—H16	118.4	N3—O2—Ag1^i^	143.5 (4)
C22—C21—C26	118.8 (7)	Ag2^ii^—O2—Ag1^i^	98.69 (18)
C22—C21—P1	123.8 (5)	N4—O4—Ag1^iii^	116.8 (5)
C26—C21—P1	117.4 (6)	N4—O4—Ag2	101.4 (4)
C21—C22—C23	120.1 (7)	Ag1^iii^—O4—Ag2	93.95 (17)
C21—C22—H22	120.0	N1—P1—C11	104.8 (3)
C23—C22—H22	120.0	N1—P1—C21	108.9 (3)
C24—C23—C22	120.1 (7)	C11—P1—C21	102.0 (3)
C24—C23—H23	120.0	N1—P1—Ag1	114.0 (2)
C22—C23—H23	120.0	C11—P1—Ag1	113.7 (2)
C23—C24—C25	121.1 (7)	C21—P1—Ag1	112.5 (2)
C23—C24—H24	119.4	N2—P2—C31	103.9 (3)
C25—C24—H24	119.4	N2—P2—C41	111.0 (3)
C24—C25—C26	118.8 (7)	C31—P2—C41	101.4 (3)
C24—C25—H25	120.6	N2—P2—Ag2	118.8 (2)
C26—C25—H25	120.6	C31—P2—Ag2	106.6 (2)
C21—C26—C25	121.0 (7)	C41—P2—Ag2	113.0 (2)
C21—C26—H26	119.5	O1—Ag1—P1	164.66 (13)
C25—C26—H26	119.5	O1—Ag1—O4^i^	71.71 (19)
C32—C31—C36	118.8 (7)	P1—Ag1—O4^i^	116.31 (14)
C32—C31—P2	121.6 (5)	O1—Ag1—O2^iii^	67.27 (17)
C36—C31—P2	118.6 (5)	P1—Ag1—O2^iii^	126.75 (13)
C31—C32—C33	119.7 (7)	O4^i^—Ag1—O2^iii^	72.66 (17)
C31—C32—H32	120.2	O2^iv^—Ag2—P2	154.16 (13)
C33—C32—H32	120.2	O2^iv^—Ag2—O4	75.92 (19)
C32—C33—C34	120.8 (8)	P2—Ag2—O4	118.77 (12)
C32—C33—H33	119.6	O2^iv^—Ag2—O1^iii^	66.42 (17)
C34—C33—H33	119.6	P2—Ag2—O1^iii^	137.62 (12)
C35—C34—C33	118.4 (8)	O4—Ag2—O1^iii^	66.56 (16)
C35—C34—H34	120.8		
			
C16—C11—C12—C13	5.3 (12)	C12—C11—P1—N1	90.4 (6)
P1—C11—C12—C13	−174.2 (7)	C16—C11—P1—C21	24.5 (9)
C11—C12—C13—C14	−5.7 (14)	C12—C11—P1—C21	−156.0 (6)
C12—C13—C14—C15	2.9 (17)	C16—C11—P1—Ag1	145.8 (8)
C13—C14—C15—C16	0(2)	C12—C11—P1—Ag1	−34.7 (7)
C14—C15—C16—C11	0(2)	C22—C21—P1—N1	10.2 (7)
C12—C11—C16—C15	−2.4 (16)	C26—C21—P1—N1	−167.9 (6)
P1—C11—C16—C15	177.0 (10)	C22—C21—P1—C11	−100.2 (7)
C26—C21—C22—C23	−4.0 (12)	C26—C21—P1—C11	81.7 (6)
P1—C21—C22—C23	177.9 (7)	C22—C21—P1—Ag1	137.6 (6)
C21—C22—C23—C24	3.8 (14)	C26—C21—P1—Ag1	−40.5 (6)
C22—C23—C24—C25	−0.8 (14)	N1—N2—P2—C31	−150.4 (5)
C23—C24—C25—C26	−1.8 (13)	C3—N2—P2—C31	56.0 (6)
C22—C21—C26—C25	1.3 (12)	N1—N2—P2—C41	101.4 (5)
P1—C21—C26—C25	179.6 (6)	C3—N2—P2—C41	−52.2 (7)
C24—C25—C26—C21	1.6 (13)	N1—N2—P2—Ag2	−32.2 (5)
C36—C31—C32—C33	3.8 (11)	C3—N2—P2—Ag2	174.2 (5)
P2—C31—C32—C33	172.1 (6)	C32—C31—P2—N2	29.7 (7)
C31—C32—C33—C34	−3.1 (12)	C36—C31—P2—N2	−162.0 (5)
C32—C33—C34—C35	2.7 (13)	C32—C31—P2—C41	144.9 (6)
C33—C34—C35—C36	−3.2 (13)	C36—C31—P2—C41	−46.8 (6)
C34—C35—C36—C31	4.0 (13)	C32—C31—P2—Ag2	−96.6 (6)
C32—C31—C36—C35	−4.2 (11)	C36—C31—P2—Ag2	71.7 (6)
P2—C31—C36—C35	−172.8 (6)	C42—C41—P2—N2	−118.9 (5)
C46—C41—C42—C43	1.3 (10)	C46—C41—P2—N2	64.1 (6)
P2—C41—C42—C43	−175.9 (5)	C42—C41—P2—C31	131.1 (5)
C41—C42—C43—C44	−2.9 (11)	C46—C41—P2—C31	−45.8 (6)
C42—C43—C44—C45	4.3 (12)	C42—C41—P2—Ag2	17.4 (6)
C43—C44—C45—C46	−4.0 (12)	C46—C41—P2—Ag2	−159.5 (5)
C44—C45—C46—C41	2.4 (11)	N3—O1—Ag1—P1	20.1 (9)
C42—C41—C46—C45	−1.0 (10)	Ag2^i^—O1—Ag1—P1	164.7 (4)
P2—C41—C46—C45	175.9 (6)	N3—O1—Ag1—O4^i^	−103.9 (5)
C2—C1—N1—N2	82.7 (10)	Ag2^i^—O1—Ag1—O4^i^	40.72 (17)
C2—C1—N1—P1	−101.0 (9)	N3—O1—Ag1—O2^iii^	177.7 (5)
C1—N1—N2—C3	71.4 (8)	Ag2^i^—O1—Ag1—O2^iii^	−37.63 (16)
P1—N1—N2—C3	−105.2 (6)	N1—P1—Ag1—O1	60.8 (6)
C1—N1—N2—P2	−83.9 (7)	C11—P1—Ag1—O1	−179.2 (6)
P1—N1—N2—P2	99.5 (5)	C21—P1—Ag1—O1	−63.9 (6)
C4—C3—N2—N1	68.1 (8)	N1—P1—Ag1—O4^i^	179.4 (3)
C4—C3—N2—P2	−138.0 (6)	C11—P1—Ag1—O4^i^	−60.6 (3)
O3—N3—O1—Ag1	−16.4 (8)	C21—P1—Ag1—O4^i^	54.7 (3)
O2—N3—O1—Ag1	165.2 (5)	N1—P1—Ag1—O2^iii^	−93.2 (3)
O3—N3—O1—Ag2^i^	−145.2 (5)	C11—P1—Ag1—O2^iii^	26.8 (3)
O2—N3—O1—Ag2^i^	36.3 (9)	C21—P1—Ag1—O2^iii^	142.1 (3)
O3—N3—O2—Ag2^ii^	−7.7 (9)	N2—P2—Ag2—O2^iv^	91.3 (4)
O1—N3—O2—Ag2^ii^	170.8 (4)	C31—P2—Ag2—O2^iv^	−151.9 (4)
O3—N3—O2—Ag1^i^	−169.2 (6)	C41—P2—Ag2—O2^iv^	−41.4 (4)
O1—N3—O2—Ag1^i^	9.2 (11)	N2—P2—Ag2—O4	−148.9 (3)
O6—N4—O4—Ag1^iii^	−67.3 (9)	C31—P2—Ag2—O4	−32.1 (3)
O5—N4—O4—Ag1^iii^	114.0 (6)	C41—P2—Ag2—O4	78.4 (3)
O6—N4—O4—Ag2	−167.7 (8)	N2—P2—Ag2—O1^iii^	−63.0 (3)
O5—N4—O4—Ag2	13.6 (7)	C31—P2—Ag2—O1^iii^	53.8 (3)
N2—N1—P1—C11	−141.4 (5)	C41—P2—Ag2—O1^iii^	164.3 (3)
C1—N1—P1—C11	42.3 (7)	N4—O4—Ag2—O2^iv^	85.6 (4)
N2—N1—P1—C21	110.1 (5)	Ag1^iii^—O4—Ag2—O2^iv^	−32.81 (18)
C1—N1—P1—C21	−66.2 (7)	N4—O4—Ag2—P2	−71.5 (4)
N2—N1—P1—Ag1	−16.4 (5)	Ag1^iii^—O4—Ag2—P2	170.14 (9)
C1—N1—P1—Ag1	167.2 (6)	N4—O4—Ag2—O1^iii^	155.6 (5)
C16—C11—P1—N1	−89.0 (8)	Ag1^iii^—O4—Ag2—O1^iii^	37.26 (16)

Symmetry codes: (i) −*x*, −*y*, *z*+1/2; (ii) *x*, *y*, *z*+1; (iii) −*x*, −*y*, *z*−1/2; (iv) *x*, *y*, *z*−1.
